# Lecanemab Treatment in a Specialty Memory Clinic

**DOI:** 10.1001/jamaneurol.2025.1232

**Published:** 2025-05-12

**Authors:** Madeline Paczynski, Anna Hofmann, Zachary Posey, Maren Gregersen, Michelle Rudman, Dawn Ellington, Melissa Aldinger, Erik S. Musiek, David M. Holtzman, Randall J. Bateman, Justin M. Long, Nupur Ghoshal, David B. Carr, Alan Dow, Sheyda Namazie-Kummer, Nayid Jana, Chengjie Xiong, John C. Morris, Tammie L. S. Benzinger, Suzanne E. Schindler, B. Joy Snider

**Affiliations:** 1 Department of Neurology, Washington University School of Medicine in St Louis, St Louis, Missouri; 2 Knight Alzheimer Disease Research Center, Washington University School of Medicine in St Louis, St Louis, Missouri; 3 Department of Medicine, Washington University School of Medicine in St Louis, St Louis, Missouri; 4 BJC Healthcare, St Louis, Missouri; 5 Department of Radiology, Washington University School of Medicine in St Louis, St Louis, Missouri; 6 Division of Biostatistics, Washington University in St Louis, St Louis, Missouri

## Abstract

**Importance:**

Two monoclonal antibodies targeting amyloid plaques, lecanemab and donanemab, have received traditional US Food and Drug Administration (FDA) approval for the treatment of early symptomatic Alzheimer disease (AD). The most significant adverse events associated with these therapies are infusion-related reactions and amyloid-related imaging abnormalities (ARIA) with edema/effusion (ARIA-E) and/or hemorrhage/hemosiderin deposition (ARIA-H). The feasibility and safety of providing these treatments in clinical practice is unclear.

**Objective:**

To examine the feasibility and safety of treating patients in specialty memory clinics with lecanemab.

**Design, Setting, and Participants:**

This retrospective analysis of consecutive patients in whom lecanemab was initiated between August 1, 2023, and October 1, 2024, at Washington University Memory Diagnostic Center, an outpatient specialty memory clinic. Lecanemab was initiated in 234 patients with early symptomatic AD. Eligibility was based on the FDA label and appropriate use recommendations with occasional exceptions.

**Exposure:**

Patients were treated with lecanemab, 10 mg/kg, intravenously every 2 weeks.

**Main Outcomes and Measures:**

Infusion-related reactions, ARIA, and withdrawal from treatment were assessed.

**Results:**

The 234 patients treated with lecanemab had a mean age of 74.4 (SD, 6.7) years, 117 were female (50%), and 117 were male (50%). Infusion-related reactions occurred in 87 patients (37%) and were typically mild. Of the 194 patients at risk for ARIA during the study period, 44 had at least 1 microhemorrhage and/or superficial siderosis before initiation of lecanemab (23%). Over an average treatment period of 6.5 months, 42 total patients (22%) developed ARIA; 29 developed ARIA-E with or without ARIA-H (15%) and 13 developed isolated ARIA-H (6.7%). Eleven patients (5.7%) developed symptomatic ARIA, 2 of those patients (1.0%) with clinically severe symptoms. No patients developed a macrohemorrhage or died. Patients with mild dementia had a 27% rate of symptomatic ARIA; those with mild cognitive impairment or very mild dementia had a 1.8% rate. Overall, 23 of 234 patients (9.8%) withdrew from treatment for various reasons, 10 for ARIA (4.3%).

**Conclusions and Relevance:**

A single-specialty memory clinic initiated lecanemab treatment in 234 patients over 14 months. The frequency of significant adverse events, including ARIA, was manageable. These results may inform discussions about the risks of anti-amyloid treatments.

## Key Points

**Question:**

Is it feasible and safe to treat patients with early symptomatic Alzheimer disease with lecanemab in a specialty memory clinic setting?

**Findings:**

In this study, lecanemab treatment was initiated in 234 patients over 14 months; infusion-related reactions were common (37%) and typically mild. Amyloid-related imaging abnormalities were observed in 42 of 194 patients (22%) who received at least 4 lecanemab infusions and underwent at least 1 monitoring magnetic resonance imaging, including 11 (5.7%) with symptomatic amyloid-related imaging abnormalities and 2 (1.0%) with clinically severe amyloid-related imaging abnormalities.

**Meaning:**

In this study, treatment with lecanemab was feasible in a specialty memory clinic and the frequency of significant adverse events was manageable.

## Teaser

This study examines the feasibility and safety of treating patients in specialty memory clinics with lecanemab.

## Introduction

Alzheimer disease (AD) remains a leading cause of death and disability globally with no cure.^1,2^ Treatments, such as acetylcholinesterase inhibitors and *N*-methyl-D-aspartate receptor antagonists, are associated with mild symptomatic benefits but do not modify the overall disease course.^3^ In contrast, 2 phase 3 clinical trials demonstrated that the anti-amyloid antibodies lecanemab^4^ and donanemab^5^ lower levels of amyloid plaques and slow cognitive decline in individuals with early symptomatic AD, which encompasses mild cognitive impairment (MCI), very mild dementia, and mild dementia due to AD. Lecanemab became the first disease-modifying treatment for AD to receive traditional approval from the US Food and Drug Administration (FDA) in July 2023^6^ and donanemab received traditional FDA approval in July 2024.^7^

Treating patients with anti-amyloid antibodies requires expertise in diagnosing early symptomatic AD and infrastructure to provide infusions and monitor for and manage adverse effects. There has been concern about the feasibility of safely providing anti-amyloid antibodies in clinical practice, although early data suggests it is possible.^8^ The most significant potential adverse effect of anti-amyloid antibodies is amyloid-related imaging abnormalities with edema/effusion (ARIA-E) or hemorrhage/hemosiderin deposition (ARIA-H), which includes microhemorrhages and superficial siderosis.^9,10,11,12,13^ ARIA is usually asymptomatic but can be associated with clinical symptoms, such as headache, visual disturbance, confusion, or focal neurological deficits, as was observed in 2.8% of participants in the CLARITY-AD trial of lecanemab^4^ and in 6.1% of those in the TRAILBLAZER-ALZ 2 trial of donanemab.^5^ Additionally, of 1612 individuals treated with lecanemab in the core or open-label extension of CLARITY-AD, 4 deaths (0.2% of individuals treated) were deemed potentially related to study medication.^4,14^ Additional deaths related to anti-amyloid antibodies have been reported in the FDA Adverse Event Reporting System, although the exact numbers are difficult to discern because the same cases are sometimes reported multiple times.^15^ As anti-amyloid antibodies move from clinical trials, where rigorous inclusion/exclusion criteria are applied and safety and monitoring protocols are closely followed, to clinical practice, where clinical judgement may widen inclusion criteria and protocols may be less rigorously followed, the safety of treatments must be carefully examined.

The Memory Diagnostic Center (MDC) is an academic specialty memory clinic associated with Barnes-Jewish Hospital (BJC) and Washington University School of Medicine that evaluates and provides longitudinal care for over 3500 patients with cognitive concerns annually. Anticipating the FDA approval of anti-amyloid treatments, MDC and BJC leadership met regularly with colleagues across multiple disciplines from 2022 through 2023 to build capacity for clinical visits, AD biomarker testing, brain imaging, and infusions. In the current study, the feasibility and safety of providing lecanemab therapy to MDC patients was examined.

## Methods

### Study Population and Workflow

Consecutive patients who received at least 1 lecanemab infusion ordered by MDC clinicians prior to October 1, 2024, were included in this analysis. The study period, including clinical follow-up for ARIA and cognitive assessments, was extended until December 1, 2024, to enable follow-up after lecanemab initiation.

Patients were initially evaluated by 1 of 14 physicians (13 neurologists and 1 geriatrician) experienced in the diagnosis of symptomatic AD. Patients identified as potential candidates for lecanemab based on the FDA label and appropriate use recommendations (AUR)^6,16^ were referred to the newly established MDC treatment clinic where they were managed by an advanced practice professional (APP) who coordinated care with the referring physician.

Potential candidates for anti-amyloid antibodies underwent a comprehensive evaluation, including cognitive testing (eAppendix 1 in Supplement 1 for specific tests), routine laboratory tests, a brain magnetic resonance imaging (MRI), biomarker testing for amyloid pathology, and apolipoprotein E (APOE) genotyping. Additionally, the severity of cognitive impairment was rated with the Clinical Dementia Rating (CDR) and CDR Sum of Boxes (CDR-SB). All candidates for treatment had a diagnosis of early symptomatic AD with a CDR of 0.5 (MCI or very mild dementia) or 1 (mild dementia)^17^; most had a Mini Mental State Examination (MMSE)^18^ score of 22 or higher.

Patients with conditions that would have excluded them from treatment based on AUR^16^ were considered on a case-by-case basis and sometimes discussed at a monthly MDC clinical conference. These cases included patients taking anticoagulant medications and patients with mild dementia but disproportionately poor performance on cognitive tests (eg, MMSE score less than 22) due to aphasia. Complex cases were typically discussed with multiple MDC clinicians including the MDC Director (B.J.S.). Final decisions to initiate treatment were made by individual clinicians after extensive discussions about the risks and benefits of treatment with patients and their families.

Lecanemab was administered via intravenous infusion every 2 weeks at infusion centers. As recommended by the FDA label,^6^ patients underwent monitoring MRIs before the fifth, seventh, and fourteenth infusion. MDC staff informed the physician–APP team when monitoring MRIs were available for review. Adverse events were documented and managed by the physician–APP team with oversight from the MDC Director (B.J.S.).

A total of 5 full-time equivalent (FTE) staff were required for the treatment clinic: 1.5 FTE APPs, 1 FTE nurse, 1 FTE treatment coordinator, 1 FTE medical assistant, and 0.5 FTE cognitive tester. MDC staff managed insurance approvals and coordinated scheduling of infusions and monitoring MRIs. An on-call clinician rotation was established for after-hours concerns, including infusion-related reactions and potential adverse effects. MDC clinicians met with the leadership of the Washington University Department of Emergency Medicine to discuss management of patients receiving anti-amyloid antibodies. Within the patients’ electronic health records, an alert was added indicating a contraindication to thrombolytic agents, as well as a requirement for an override to complete an order for thrombolytics.^19^

### Biomarker Testing and APOE Genotyping

All patients underwent biomarker testing to confirm the presence of amyloid pathology. Cerebrospinal fluid (CSF) testing used the Roche Elecsys p-tau181/Aβ42 test at either the Mayo Clinic Laboratories, Rochester, Minnesota, or the BJC Clinical Laboratory, St Louis, Missouri.^20,21^ Blood testing used the PrecivityAD2 test, which incorporates p-tau217/nonphosphorylated tau (%p-tau217) and Aβ42/Aβ40, at C2N Diagnostics in St. Louis, Missouri.^22,23^ Amyloid positron emission tomography (PET) scans were performed with multiple FDA–approved radiotracers within the BJC network.^24,25^

Multiple tests were used to assess for the presence and number of APOE ε4 alleles, including tests offered by Mayo Clinic Laboratories, EmpowerDX, 23andMe, and C2N Diagnostics. The number of APOE ε4 alleles, rather than APOE genotype, was provided by 23andMe and APOE prototyping was performed by C2N Diagnostics.

### MRI

All patients underwent a pretreatment (baseline) brain MRI to detect radiological findings known to affect risk for ARIA, such as microhemorrhages, superficial siderosis, infarcts, and severity of white matter disease, as assessed by the Fazekas score.^9,13,26^ Most baseline MRI scans were performed at Washington University/BJC–affiliated hospitals on a 3-T scanner with T1, T2, fluid-attenuated inversion recovery, diffusion weighted imaging, and susceptibility weighted imaging (SWI) sequences using American Society of Neuroradiology (ASNR) protocols.^10^ MRI scans performed at outside facilities with different scanner strengths and sequences were reviewed by the treating clinician and a Washington University neuroradiologist. If the image quality or sequences performed did not meet ASNR standards,^10^ a second scan was performed at a BJC–affiliated facility. For baseline MRI scans, data are presented on the original read that informed clinical decision-making.

All follow-up MRI scans were performed at BJC–affiliated facilities, typically with a 1.5-T scanner. The presence and number of microhemorrhages, superficial siderosis, and ARIA-E were assessed using all MRI sequences, including SWI and gradient-recalled echo/T2*, as per ASNR guidelines.^10^ For patients who developed ARIA, data are presented on the scan with the maximum ARIA severity for that patient over their entire course and final reads were performed by Benzinger and her team. Assessment of the ARIA severity was categorized consistent with FDA guidelines.^10^

### Clinical Assessment of Infusion-Related Reactions and ARIA

Infusion-related reactions were graded according to the Common Terminology Criteria for Adverse Events.^27^ Symptoms of ARIA were assessed in patients who reported symptoms or who had ARIA found on monitoring MRIs and the clinical severity was classified according to previously established categories.^16^

### Clinical Data Acquisition and Approvals

Reporting guidelines for a case series were followed as recommended by Kempen et al.^28^ The characteristics of patients (demographics and cognitive data, number of APOE ε4 alleles, history of comorbidities, and medications) treated with lecanemab were collected by the MDC treatment clinic and reviewed by clinicians, whereas information on the larger MDC cohort was extracted from electronic health records. Potential infusion-related reactions and symptoms of ARIA were assessed by review of the electronic health records and, if needed, further investigation by an MDC clinician.

Approval for this study was obtained from the Washington University institutional review board. For the 11 patients with symptomatic ARIA, written consent was obtained to enable detailed description of their cases.

### Statistical Analyses and Data Visualization

The significance of differences was assessed with *t* tests for continuous variables, Mantel-Haenszel tests for ordinal variables, and Fisher exact tests for binary variables. To estimate the rate of change in CDR-SB, linear mixed-effect models with random slopes and intercepts were implemented. Missing data for race, ethnicity, APOE genotype, and mean arterial blood pressure were not included in comparisons. All tests were 2-sided and were not adjusted for multiple comparisons. Statistical analyses were implemented using SAS version 9.4 (SAS Institute). Plots were created with Prism version 10.4.0 (GraphPad) or SAS version 9.4.

## Results

### Characteristics of MDC Patients Receiving Lecanemab

Based on electronic health record data, an estimated 3938 unique patients were seen at the MDC between August 1, 2023, and October 1, 2024 (eTable 1 in Supplement 1). Both a CDR and MMSE score were entered into the electronic health records for 2670 patients; 1452 had a documented CDR of 0.5 or 1 and an MMSE score of 22 or higher. Treatment with lecanemab was initiated in 234 MDC patients (5.9% of total patients seen). Compared with treated participants in the CLARITY-AD trial, MDC patients treated with lecanemab were slightly older (74.4 [SD, 6.7] years vs 71.4 [SD, 7.9] years; *P* < .001) and had lower MMSE scores (24 [SD, 4] vs 26 [SD, 2]; *P* < .001; eTable 1 in Supplement 1). Half of MDC patients treated with lecanemab were female and nearly all identified as White (98%). Only 3 patients identified as Black (1.3%). A lower percentage of MDC patients treated with lecanemab were APOE ε4 homozygotes compared with the CLARITY-AD study (8.5% vs 16%, *P* = .004; eTable 1 in Supplement 1). Nine patients (3.8%) were receiving anticoagulant medications (apixaban or rivaroxaban) and 83 patients (35%) were taking antiplatelet agents (aspirin and/or clopidogrel).

Most patients underwent CSF testing (139 [59%]) or amyloid PET (55 [24%]) for confirmation of amyloid pathology. High-accuracy blood tests were performed in 40 patients (17%); in 37 patients, the high-accuracy blood test was the only AD biomarker test performed.^29^ Notably, many patients underwent biomarker testing before the cost of amyloid PET scans was covered by the US Centers for Medicare and Medicaid Services.

### Infusion-Related Reactions

Infusion-related reactions occurred in 87 of 234 patients (37%) and were more frequent than reported in the CLARITY-AD trial (26%; *P* = .002). Most infusion-related reactions occurred following the first or second infusion (92%) and were usually rated as mild (67%) or moderate (28%). Commonly reported symptoms included chills, headache, nausea, and fever. Because of the high rate of infusion-related reactions, the treatment protocol was changed in mid-July 2024 to include pretreatment with an antihistamine (loratadine) and acetaminophen before initial infusions; after this change, the rate of infusion-related reactions decreased to 27% (10 of 37). Occasional patients were treated with steroids, such as a patient who developed a skin rash following infusions.

Five of 234 patients (2.1%) developed more severe or atypical infusion-related reactions with symptoms that started within 24 hours of the infusion: 3 patients developed weakness and had a fall requiring hospitalization after their first infusion; 1 patient developed a thrombotic microangiopathy with severe acute kidney injury that resolved with hospitalization, plasmapheresis, steroids, and supportive care; and 1 patient developed throbbing leg pain following each infusion. In all 5 patients, urgent MRI scans showed no evidence of ARIA.

### ARIA

No patients with fewer than 4 infusions developed symptomatic ARIA or had ARIA findings on urgent MRI scans. Therefore, the 194 patients who received at least 4 lecanemab infusions and underwent at least 1 monitoring MRI were considered at risk for ARIA during the study period. Importantly, on the pretreatment scans, a total of 44 patients (23%) had at least 1 microhemorrhage and/or superficial siderosis even before lecanemab treatment was initiated: 42 patients (22%) had at least 1 microhemorrhage and 7 patients (3.6%) had at least 1 focus of superficial siderosis (Table 1).

**Table 1.** Baseline Characteristics of Patients With and Without Amyloid-Related Imaging Abnormalities (ARIA)

Characteristic	No. (%)							
	All at risk for ARIA (n = 194)^a^	No ARIA (n = 152)^b^	ARIA-E [with or without ARIA-H] (n = 29)	*P* value	Isolated ARIA-H (n = 13)	*P* value	Any ARIA (n = 42)	*P* value
Mean age (SD), y	74.3 (6.7)	74.5 (6.7)	73.7 (6.4)	.76	73.8 (7.9)	.37	73.7 (6.8)	.92
Sex								
Female	101 (52)	77 (51)	19 (66)	.16	5 (38)	.57	24 (57)	.49
Male	93 (48)	75 (49)	10 (33)		8 (62)		18 (43)	
MMSE score (SD)	24 (4)	24 (4)	22 (5)	.009	24 (4)	.71	23 (5)	.02
Clinical dementia rating								
0.5	164 (85)	131 (86)	20 (69)	.03	13 (100)	.38	33 (79)	.23
1	30 (15)	21 (14)	9 (31)		0 (0)		9 (21)	
Race and ethnicity^c^								
Asian	1 (0.5)	1 (0.7)	0	.99	0	.41	0	.77
Black	2 (1.0)	2 (1.3)	0		0		0	
Hispanic	3 (1.5)	2 (1.3)	0		1 (7.7)		1 (2.4)	
White	190 (98)	148 (97)	29 (100)		13 (100)		42 (100)	
Unknown	1 (0.5)	1 (0.7)	0	NA	0	NA	0	NA
APOE ε4 status								
ε4 Homozygote	16 (8.2)	9 (6.0)	6 (21)	.02	1 (7.7)	.23	7 (17)	.01
ε4 Heterozygote	102 (53)	78 (52)	15 (52)		9 (69)		24 (57)	
ε4 Non-carrier	74 (38)	63 (42)	8 (28)		3 (23)		11 (26)	
Unknown	2 (1.0)	2 (1.3)	0	NA	0	NA	0	NA
Mean arterial blood pressure, mm Hg								
<93	63 (32)	47 (31)	13 (45)	.74	3 (23)	.47	16 (38)	.94
93 to <97	27 (14)	22 (15)	2 (6.9)		3 (23)		5 (12)	
97 to <107	68 (35)	59 (39)	7 (24)		2 (15)		9 (21)	
>107	31 (16)	20 (13)	7 (24)		4 (31)		11 (26)	
Missing	5 (2.6)	4 (2.6)	0	NA	1 (8)	NA	1 (2.3)	NA
Comorbidities								
Hypertension	119 (61)	94 (62)	18 (62)	.99	7 (54)	.57	25 (60)	.86
Dyslipidemia	161 (83)	127 (84)	24 (83)	.99	10 (77)	.46	34 (81)	.65
Diabetes	21 (11)	15 (9.9)	3 (10)	.99	3 (23)	.15	6 (14)	.41
Medication use								
Antiplatelet	68 (35)	53 (35)	9 (31)	.83	6 (46)	.55	15 (36)	.99
Anticoagulant	9 (4.6)	8 (5.3)	1 (3.4)	.99	0 (0)	.99	1 (2.4)	.69
Antihypertensive	81 (42)	63 (41)	13 (45)	.84	5 (38)	.99	18 (43)	.86
Baseline No. of microhemorrhages^d^								
0	152 (78)	122 (80)	23 (79)	.67	7 (54)	.01	30 (71)	.11
1-4	39 (20)	29 (19)	5 (17)		5 (38)		10 (24)	
5-9	3 (1.5)	1 (0.7)	1 (3.4)		1 (7.7)		2 (4.8)	
Baseline No. of superficial siderosis foci^d^								
0	187 (96)	148 (97)	27 (93)	.42	12 (92)	.48	39 (93)	.32
1	6 (3.1)	3 (2.0)	2 (6.9)		1 (7.7)		3 (7.1)	
2	1 (0.5)	1 (0.7)	0		0		0	
Baseline Fazekas score								
0	26 (13)	20 (13)	6 (21)	.89	0	.40	6 (14)	.75
1	126 (65)	100 (66)	15 (52)		11 (85)		26 (62)	
2	40 (21)	31 (20)	8 (28)		1 (7.7)		9 (21)	
3	2 (1.0)	1 (0.7)	0		1 (7.7)		1 (2.4)	
Baseline infarcts								
≥1 Small infarcts (<10 mm)	22 (11)	14 (9.2)	5 (17)	.20	3 (23)	.14	8 (19)	.10
1 Medium-sized infarct (10-20 mm)	2 (1.0)	0	1 (3.4)	.16	1 (7.7)	.08	2 (4.8)	.05

Abbreviations: APOE, apolipoprotein E; ARIA-E, ARIA with edema/effusion; ARIA-H, hemorrhage hemosiderin deposition; MMSE, Mini-Mental State Examination; NA, not applicable due to missing data.

^a^ The 194 patients who received at least 4 lecanemab infusions and 1 monitoring magnetic resonance imaging were considered at risk for ARIA.

^b^ Statistical comparisons are with the No ARIA group.

^c^ For race and ethnicity, categories may equal more than 100% due to some participants identifying with multiple groups. Race and ethnicity were self-reported.

^d^ A total of 44 patients had at least 1 microhemorrhage and/or superficial siderosis before treatment initiation.

**Figure 1.**
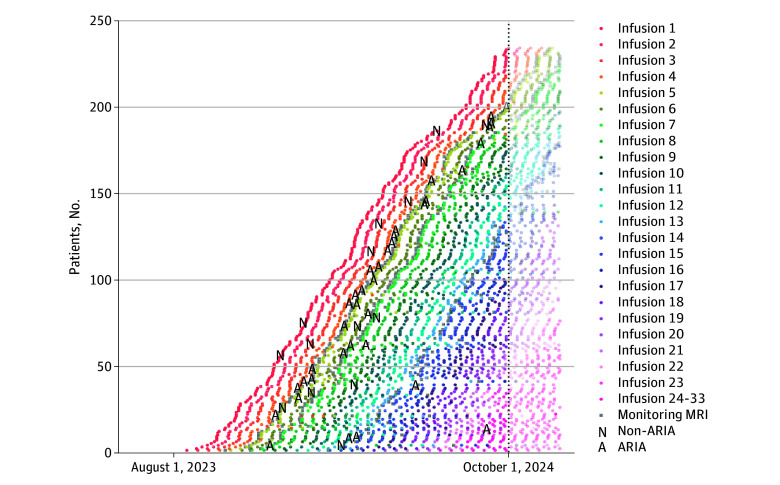
Lecanemab Infusions, Monitoring Magnetic Resonance Imaging (MRI) Scans, and Amyloid-Related Imaging Abnormalities (ARIA) Over Time Each circle indicates 1 infusion with the color denoting the number of the infusion. A indicates ARIA; N, non–ARIA event, eg, non–ARIA related issues, such as a fall.

**Figure 2.**
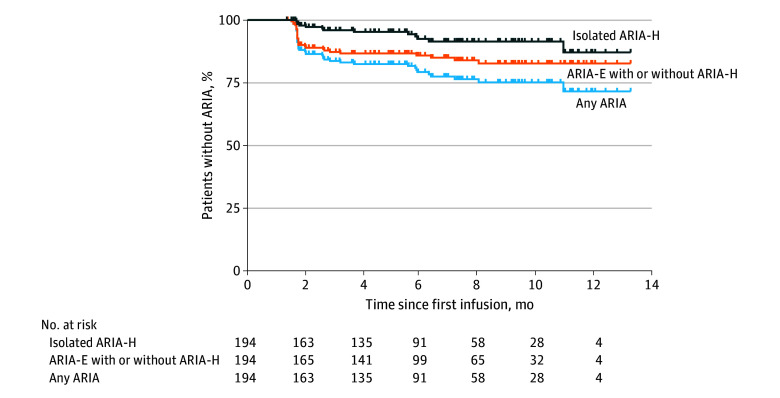
Percent of Patients With Amyloid-Related Imaging Abnormalities (ARIA) Over the First 14 Months of Treatment The analyses include only the 194 individuals at risk for ARIA who received at least 4 infusions of lecanemab and underwent at least 1 monitoring magnetic resonance imaging. The proportion of patients without isolated ARIA-H, ARIA with edema/effusion (ARIA-E) with or without ARIA with hemorrhage hemosiderin deposition (ARIA-H), or any ARIA is shown as a function of the time since first infusion. The average length of treatment was 6.5 months (see eTable 2 in Supplement 1 for length of follow-up).

The 194 patients at risk for ARIA were treated for an average of 6.5 (SD, 3.2) months during the study period (Figure 1; eTable 2 in Supplement 1). A total of 42 patients developed at least 1 ARIA finding (22%): 29 had ARIA-E with or without ARIA-H (15%) and 13 had isolated ARIA-H (6.7%) (Figure 2; Table 1). Episodes of ARIA-E with or without ARIA-H typically occurred very early in treatment (11% by 2 months and 13% by 4 months) and then rarely occurred after 6 months of treatment. In contrast, isolated ARIA-H slowly accrued over time (2% by 2 months and 5% by 4 months). Only 33 patients had follow-up longer than 10 months; the proportion of patients with an ARIA finding at 12 months of treatment was 29%: 17% with ARIA-E with or without ARIA-H and 13% with isolated ARIA-H.

Rates of ARIA-E with or without ARIA-H were associated with the number of APOE ε4 alleles: 38% for APOE ε4 homozygotes, 15% for heterozygotes, and 11% for noncarriers (eTable 3 in Supplement 1). In contrast, isolated ARIA-H was not associated with the number of APOE ε4 alleles. However, isolated ARIA-H was more frequent in patients with higher numbers of microhemorrhages at baseline (Table 1). Neither ARIA-E with or without ARIA-H or isolated ARIA-H were significantly associated with a variety of clinical factors: mean arterial blood pressure; a clinical history of hypertension, dyslipidemia, or diabetes; use of antiplatelet medications, anticoagulant medications, or antihypertensive medications. There was a trend toward a higher rate of ARIA in patients with medium-sized infarcts (Table 1).

**Figure 3.**
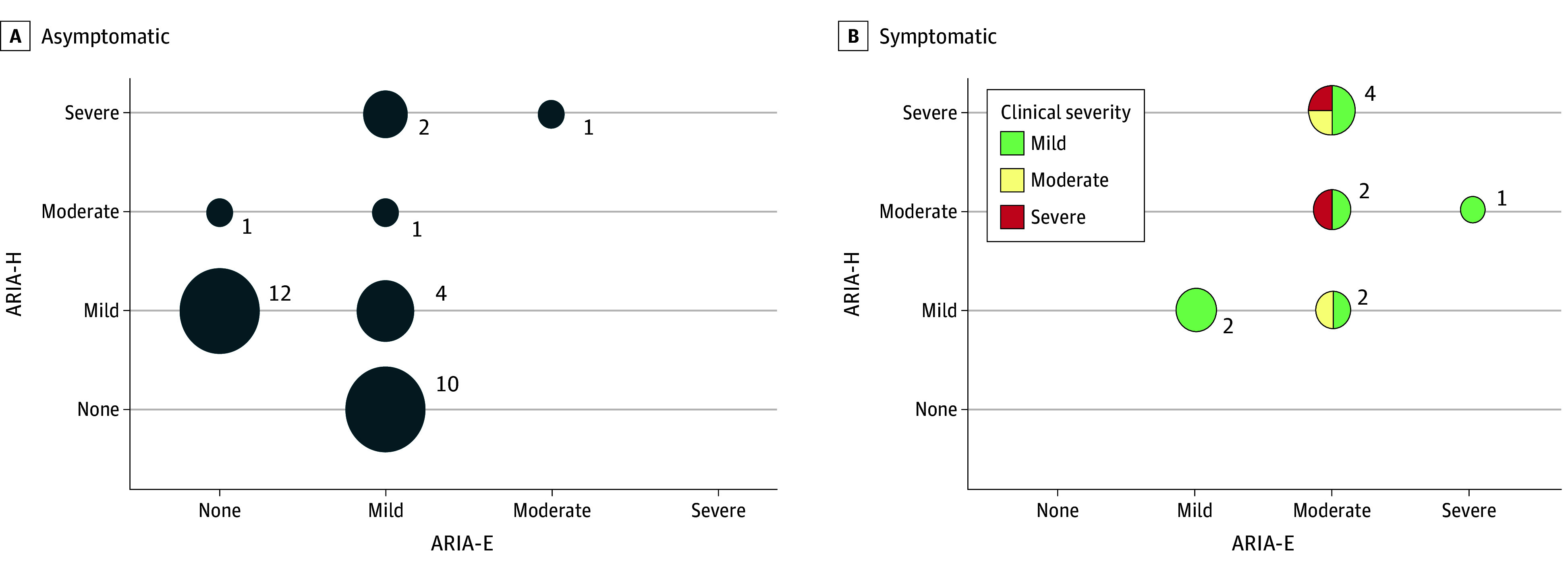
Radiographic and Clinical Severity of Amyloid-Related Imaging Abnormalities (ARIA) Cases For asymptomatic (A) and symptomatic (B) ARIA cases, the number of cases is shown according to the maximal radiographic severity of ARIA with hemorrhage hemosiderin deposition (ARIA-H) and ARIA with edema/effusion (ARIA-E). The size of the circle corresponds to the number of patients.

Most ARIA were asymptomatic (31 of 42 [74%]) and additionally radiographically mild (26 of 42 [62%]), often occurring as asymptomatic isolated mild ARIA-H or ARIA-E (22 of 42 [52%]; Figure 3; eTables 4 and 5 in Supplement 1). However, both ARIA-E and ARIA-H were present in all 11 of the patients who had symptoms associated with ARIA (Figure 3, Table 2). Representative MRI images of ARIA are shown in eFigure 1 in Supplement 1. Most cases of symptomatic ARIA (10 of 11 [91%]) were detected on the first monitoring MRI after the fourth infusion; 1 case was detected after the thirteenth infusion. Nine cases were detected on routine monitoring MRIs and patients only reported symptoms after being contacted about their MRI results. A total of 29 urgent MRIs were ordered to evaluate whether symptoms experienced by patients were associated with ARIA; only 2 demonstrated ARIA.

**Table 2.** Patients With Symptomatic Amyloid-Related Imaging Abnormalities (ARIA)^a^

Sex/age, y	Baseline CDR	APOE	Meds	Baseline Fazekas	Baseline MCH/SS	Inf No.	ARIA-E	ARIA-H	Symptoms	Clinical severity	Outcomes
F/early 70s	1	ε3/ε3	No AC/AP	2	3 MCH 0 SS	4	Moderate	Mild MCH, severe SS	See below (patient 1)^b^	Severe	See below (patient 1)^b^
M/mid 60s	1	ε3/ε3	ASA, 325 mg	0	5 MCH 1 SS	4	Moderate	Moderate MCH, no SS	See below (patient 2)^c^	Severe	See below (patient 2)^c^
F/late 70s	1	ε3/ε4	No AC/AP	2	1 MCH 0 SS	4	Moderate	Severe MCH, severe SS	Headache, subacute decline in cognition, aphasia, visual disturbance, right arm tremor, shuffling gait	Moderate	After initial MRI, worsening moderate ARIA-E, and severe ARIA-H, on follow-up MRI 2 resolving ARIA-E and stable ARIA-H; continued improvement in aphasia and confusion
M/early 70s	1	ε4/ε4	No AC/AP	2	2 MCH 0 SS	4	Moderate	Mild MCH, no new SS	Headache, subacute decline in cognition, agitation	Moderate	After initial MRI worsening ARIA-E from mild to moderate, on follow-up MRI 2 improving ARIA-E, 1 new MCH; symptoms resolved
F/late 70s	0.5	ε4/ε4	No AC/AP	1	0 MCH 0 SS	4	Moderate	Severe MCH, no SS	Subacute decline in cognition	Mild	After initial MRI worsening ARIA-H from mild to severe, on follow-up MRI 2 resolved ARIA-E and stable ARIA-H; symptoms resolved
F/mid 70s	1	ε3/ε4	No AC/AP	1	0 MCH 0 SS	4	Moderate	Moderate MCH, no SS	Change in visual acuity and color perception	Mild	On follow-up MRI 3 resolved ARIA-E and stabilize ARIA-H; symptoms resolved
F/early 70s	0.5	ε4/ε4	ASA, 81 mg	1	0 MCH 0 SS	4	Severe	Moderate MCH, mild SS	Headache, new onset bilateral upper extremity tremor	Mild	After initial MRI worsening ARIA-E from moderate to severe and ARIA-H from mild to moderate; on follow-up MRI 4 improving ARIA-E and stable ARIA-H; symptoms resolved
F/early 60s	1	ε3/ε4	No AC/AP	2	0 MCH 0 SS	4	Moderate	Severe MCH, no SS	Headache	Mild	On follow-up MRI 4 nearly resolved ARIA-E and stable ARIA-H; ongoing management of mild headaches
F/late 70s	0.5	ε2/ε3	ASA, 81 mg	2	2 MCH 0 SS	4	Moderate	Mild MCH, no SS	Headache, dizziness, visual disturbance, agitation	Mild	On follow-up MRI 1 resolved ARIA-E and stable ARIA-H; symptoms resolved
F/late 50s	1	ε3/ε4	No AC/AP	1	0 MCH 0 SS	4	Mild	Mild MCH, no SS	Headache	Mild	On follow-up MRI 2 resolved ARIA-E and stable ARIA-H; symptoms resolved
M/mid 60s	1	ε3/ε4	ASA, 81 mg; clopidogrel, 75 mg	0	0 MCH 0 SS	13	Mild	Mild MCH, no SS	Visual disturbance	Mild	On follow-up MRI 2 resolved ARIA-E and stable ARIA-H; symptoms resolved

Abbreviations: AC, anticoagulant; AP, antiplatelet; ARIA-E, ARIA with edema/effusion; ARIA-H, hemorrhage hemosiderin deposition; ASA, acetylsalicylic acid; CDR, Clinical Dementia Rating; F, female; Inf #, the number of infusions before the detection of ARIA; M, male; MCH, microhemorrhages; Meds, any anticoagulant or antiplatelet medications; MRI, magnetic resonance imaging; SS, superficial siderosis.

^a^ Details on patients with symptomatic ARIA (n = 11) are provided. Lecanemab infusions were held in all patients with symptomatic ARIA until symptoms resolved.

^b^ Patient 1 initially reported a mild headache after infusion 4. Two weeks later, after infusions were halted, she developed a subacute worsening of cognition and psychosis, right upper-extremity weakness, and right homonymous hemianopsia. Repeat MRI revealed moderate ARIA-E, an additional 2 areas of siderosis, at least 10 new microhemorrhages and punctate acute infarcts in the cerebellum. She was hospitalized and treated with high-dose intravenous steroids for 5 days, then discharged on an oral steroid taper. The most recent MRI (2 months after her fourth infusion) showed more than 20 microhemorrhages and extensive superficial siderosis. Her headaches, visual field deficit, weakness, and psychosis have improved, but she still requires ongoing treatment with antipsychotics.

^c^ Patient 2 developed a mild headache and acute worsening of cognition and gait disturbance after infusion 4. An urgent MRI revealed 5 new microhemorrhages and moderate ARIA-E. He was hospitalized and treated with high-dose intravenous steroids for 5 days, then discharged on an oral steroid taper. The most recent MRI (about 3 months after his fourth infusion) showed resolving ARIA-E and stable ARIA-H. His symptoms resolved and his neurological examination returned to pretreatment baseline.

Patients with mild dementia (CDR of 1) at baseline had a 15-fold higher rate of symptomatic ARIA (8 of 30 [27%]) compared with patients with MCI or very mild dementia (CDR of 0.5) at baseline (3 of 164 [1.8%]); *P* < .001 (eTable 4 in Supplement 1). Additionally, patients who developed symptomatic ARIA had significantly lower baseline MMSE scores (19 [SD, 7]) compared with patients who did not develop ARIA (24 [SD, 4]; *P* < .001). Descriptions of the 11 patients who developed symptomatic ARIA are provided in Table 2. Seven of the 11 patients with symptomatic ARIA had mild symptoms that caused discomfort but no disruption of daily activities. Two patients had moderate symptoms that caused discomfort sufficient to affect their daily activities. Two patients with severe symptoms were unable to perform daily activities and were hospitalized; both had mild dementia (CDR of 1) at baseline.

In all patients with symptomatic ARIA, lecanemab infusions were held and monthly follow-up MRIs were performed until resolution of ARIA-E and stabilization of ARIA-H. After lecanemab infusions were stopped in 3 patients with symptomatic ARIA and 1 patient with asymptomatic moderate ARIA, the radiographic severity stage worsened but then later improved; 3 of these 4 patients were APOE ε4 homozygotes.

At the end of the study period on December 1, 2024, 8 of 11 patients with symptomatic ARIA had resolution of their symptoms. One patient had persistent mild aphasia and confusion, 1 patient had an exacerbation of chronic mild headaches, and 1 patient had an exacerbation of psychosis. Eight patients had radiographic improvement, typically between the second and fourth follow-up MRI (approximately 3 months after ARIA was detected). Three patients had resumed lecanemab treatment without further complications and 2 additional patients planned to resume treatment when eligible. No hemorrhages more than 1 cm or deaths were observed during the study period.

### Discontinuation of Lecanemab

Over the study period, 23 patients (9.8%) withdrew from lecanemab treatment for the following reasons: 6 for symptomatic and 4 for asymptomatic ARIA; 3 for typical and 5 for atypical infusion-related reactions; 1 for unrelated health issues; 1 for anxiety about the potential for ARIA; 1 for intolerance of MRI scans; 1 for burden of treatment and monitoring regimen; and 1 for cessation of insurance coverage. Most patients who developed ARIA did not withdraw from treatment (76%).

### Cognitive Monitoring

In preliminary analyses we assessed the rate of change in the CDR-SB (eFigure 2 in Supplement 1). In the same cohort of 194 individuals who received at least 4 lecanemab infusions and a monitoring MRI (Table 1), 155 patients had a baseline CDR-SB an average of 2.6 (SD, 1.8) months before treatment initiation and a follow-up CDR-SB an average of 7.0 (SD, 2.9) months following lecanemab initiation. The estimated rate of change over the treatment period was 1.11 CDR-SB per year, with a change of 1.56 CDR-SB per year for individuals with mild dementia (CDR of 1) at baseline and 0.99 CDR-SB per year for individuals with MCI or very mild dementia (CDR of 0.5) at baseline (eTable 6 and eAppendix 2 in Supplement 1).

## Discussion

The primary objective of this study was to evaluate the feasibility and safety of treating specialty memory clinic patients with lecanemab. Overall, we found that it is possible to treat patients with anti-amyloid antibodies with a relatively low rate of significant complications. Notably, Washington University has been a leading site for clinical trials of anti-amyloid antibodies for many years. Most MDC clinicians have significant experience in managing ARIA and Washington University neuroradiologists are experts at reading MRI scans for signs of ARIA. Even with this expertise, initiating anti-amyloid antibody treatment in 234 patients over 14 months required creating a designated treatment clinic with 5 fulltime staff. The additional clinic staff, including APPs, were essential to increasing clinical capacity and managing major logistical issues associated with the complex evaluation and treatment pathway.

A relatively small percentage of patients seen at the MDC received lecanemab (5.9%), which reflects many issues, including that most MDC patients did not meet basic clinical criteria for early symptomatic AD (eg, a CDR of of 0.5 or 1 and an MMSE score of 22 or higher). Overall, lecanemab treatment was well tolerated with discontinuation in only 9.8% of patients. Infusion-related reactions were more frequent than reported in the CLARITY-AD trial, but decreased after the treatment protocol was changed to include pretreatment with an antihistamine and acetaminophen before initial infusions. ARIA occurred in 42 of 194 patients (22%) who received at least 4 lecanemab infusions and underwent at least 1 monitoring MRI. The majority of ARIA was asymptomatic and radiographically mild, and most patients who developed ARIA did not withdraw from treatment. No deaths or macrohemorrhages were observed during the study period.

Importantly, patients with mild dementia (CDR of 1) at baseline had a 15-fold higher rate of symptomatic ARIA compared with patients with MCI or very mild dementia (CDR of 0.5) at baseline (27% vs 1.8%). Furthermore, individuals who developed symptomatic ARIA had lower baseline MMSE scores compared with individuals who did not develop ARIA. Two patients treated with lecanemab developed severe symptomatic ARIA, representing 1.0% of patients treated; both had mild dementia at baseline (CDR of 1). These findings suggest that risk for symptomatic ARIA is strongly associated with the severity of clinical symptoms, potentially because of the increasing burden of cerebral amyloid angiopathy with advancing AD,^9,12^ and underscore the importance of diagnosing and treating symptomatic AD as early as possible.

Of the 194 patients at risk for ARIA during the study period, 44 patients (23%) had at least 1 microhemorrhage and/or superficial siderosis before lecanemab treatment was initiated. Of note, patients with a greater burden of microhemorrhages and/or superficial siderosis were excluded from treatment and, therefore, 23% is an underestimate of the prevalence of these findings in patients with early symptomatic AD. This suggests that a proportion of ARIA-H that develops during the course of treatment is unrelated to anti-amyloid antibody treatment. In fact, some patients receiving placebo had ARIA-H or ARIA-E findings in both the CLARITY-AD trial of lecanemab^4^ and the TRAILBLAZER-ALZ 2 trial of donanemab.^5^ The common occurrence of ARIA findings in untreated patients is rarely discussed, but may affect the perceived significance of findings that are often described to patients as brain bleeding and brain swelling directly caused by treatments.

The rate of any ARIA findings was 22% over an average treatment period of 6.5 months. ARIA-E with or without ARIA-H typically developed shortly after initiation of lecanemab, occurred in 15% of patients, and was associated with the number of APOE ε4 alleles. In contrast, isolated ARIA-H slowly accrued over time, occurred in 6.7% of patients, was not associated with the number of APOE ε4 alleles, but was associated with the number of microhemorrhages at baseline. Importantly, MRI evaluations typically used both the susceptibility-weighted imaging and gradient echo sequences, which would be expected to increase the rate of ARIA-H detection compared with the CLARITY-AD trial that only used gradient echo.

The number of APOE ε4 alleles is known to be a risk factor for ARIA^9^ and affects the balance of risks and benefits of anti-amyloid treatments. While 16% of individuals in the CLARITY-AD trial were APOE ε4 homozygotes,^4^ only 8.5% of MDC patients treated with lecanemab were homozygotes. This may reflect that patients, families, and providers were less likely to initiate lecanemab in APOE ε4 homozygotes due to an expected higher rate of adverse events. Titration protocols, such as that recently proposed for donanemab, may further reduce the risks of anti-amyloid treatments, especially for APOE ε4 homozygotes.^30^

Although AURs currently advise against treating patients taking anticoagulant medications,^16^ the MDC treated 9 patients on anticoagulant medications who were informed of the potential risk for macrohemorrhage. No complications were observed. Unlike a recent study of donanemab that included 3030 participants,^31^ we found no association of ARIA-E with or without ARIA-H with mean arterial blood pressure, a clinical history of hypertension, or use of antihypertensive medications. However, our much smaller cohort size, low rate of ARIA, and limited follow-up period did not provide adequate power to evaluate for weaker associations with ARIA or rare but clinically severe events like macrohemorrhage.^13^

In preliminary analyses, we examined the rate of change in the CDR-SB, which was used as the primary end point in the 18-month long CLARITY-AD trial.^4^ Included patients had a baseline CDR-SB an average of 2.6 months before treatment initiation and a follow-up CDR-SB an average of 7.0 months after treatment initiation. Clearance of amyloid continues over 18 months of lecanemab treatment^4^ and most patients in the preliminary analyses would be expected to have very high amyloid burdens at the early time points shown. The estimated rate of change was over the treatment period 1.11 CDR-SB per year, which exactly corresponds with the rate of change reported for the placebo group of CLARITY-AD.^4^ Future analyses will compare change in the CDR-SB in patients treated with lecanemab for 18 months and untreated patients matched for key clinical characteristics.

### Limitations

An important concern of MDC clinicians and staff was the very low diversity of the patients being treated with lecanemab. Previous analyses have found that approximately 16% of individuals in the MDC catchment area identify as Black but only 11% of MDC patients are Black.^32^ Only 3 patients (1.3%) receiving lecanemab identified as Black. Previous work has demonstrated that Black individuals are more likely to initially present to the MDC at the moderate or severe stage of dementia when anti-amyloid treatments are no longer effective.^32^ Major structural issues in our health care system must be addressed to improve the early diagnosis of individuals who identify with racial and ethnic minoritized groups or disparities in dementia care may increase.^33^

While the MDC demonstrates how anti-amyloid antibodies can be provided in a specialty memory clinic with rich resources, it may be possible to provide these treatments in clinics with fewer resources by employing a variety of strategies, some of which are currently still in development.^34,35^ Digital cognitive assessments could improve early detection of cognitive impairment.^36,37^ The use of high accuracy blood tests could reduce the burden of AD biomarker testing.^29,38^ Electronic health record tools could facilitate efficient scheduling of infusions and MRI scans. Subcutaneous administration could substantially reduce the burden of treatment with anti-amyloid antibodies. Artificial intelligence–based assistive software tools could assist radiologists in detecting and quantifying ARIA.^39^ Telehealth consultations with more experienced clinicians could potentially help health care professionals with more complex cases or complications. As experience with anti-amyloid antibody treatments increases and new strategies are further developed, we expect that more efficient and generalizable care pathways will be created and implemented.

## Conclusions

In one specialty memory clinic, 234 patients were treated with lecanemab over 14 months. The frequency of adverse events was manageable. These findings may inform discussions about the risks of anti-amyloid treatments.

## Notes

Supplement 1. **eFigure 1.** Representative Brain MRI Scans From Patients With ARIA **eFigure 2.** Clinical Dementia Rating Sum of Boxes (CDR-SB) Over Time **eTable 1.** Characteristics of Memory Diagnostic Center (MDC) Patients and CLARITY-AD Participants **eTable 2.** Length of Treatment for Patients at Risk for ARIA **eTable 3.** Features of ARIA at Maximal Severity by Number of *APOE* ε4 Alleles **eTable 4.** Baseline Characteristics of Patients With No ARIA Compared to Asymptomatic or Symptomatic ARIA **eTable 5.** Baseline Characteristics of Patients With No ARIA Compared to Asymptomatic or Symptomatic ARIA (Continued) **eTable 6.** Rates of Change in the Clinical Dementia Rating Sum of Boxes (CDR-SB) **eAppendix 1.** Memory Diagnostic Center Cognitive Battery **eAppendix 2.** SAS Code and Model Output for Linear Mixed Models of Change in CDR-SB Over Time

Supplement 2. Data Sharing Statement
