# Supplementary material for: Lecanemab Treatment in a Specialty Memory Clinic
Source: JAMA Neurol. 2025 May 12;82(7):655–65. doi: 10.1001/jamaneurol.2025.1232 (PMC12070285; doi:10.1001/jamaneurol.2025.1232)
Supplement: Supplement 1. — eFigure 1. Representative Brain MRI Scans From Patients With ARIA eFigure 2. Clinical Dementia Rating Sum of Boxes (CDR-SB) Over Time eTable 1. Characteristics of Memory Diagnostic Center (MDC) Patients and CLARITY-AD Participants eTable 2. Length of Treatment for Patients at Risk for ARIA eTable 3. Features of ARIA at Maximal Severity by Number of APOE ε4 Alleles eTable 4. Baseline Characteristics of Patients With No ARIA Compared to Asymptomatic or Symptomatic ARIA eTable 5. Baseline Characteristics of Patients With No ARIA Compared to Asymptomatic or Symptomatic ARIA (Continued) eTable 6. Rates of Change in the Clinical Dementia Rating Sum of Boxes (CDR-SB) eAppendix 1. Memory Diagnostic Center Cognitive Battery eAppendix 2. SAS Code and Model Output for Linear Mixed Models of Change in CDR-SB Over Time [file jamaneurol-e251232-s001.pdf]

Supplemental Online Content

Paczynski M, Hofmann A, Posey Z, et al. Lecanemab treatment in a specialty memory clinic. *JAMA Neurol*. Published online May 12, 2025. doi:10.1001/jamaneurol.2025.1232

eFigure 1. Representative Brain MRI Scans From Patients With ARIA

eFigure 2. Clinical Dementia Rating Sum of Boxes (CDR-SB) Over Time

eTable 1. Characteristics of Memory Diagnostic Center (MDC) Patients and CLARITY-AD Participants

eTable 2. Length of Treatment for Patients at Risk for ARIA

eTable 3. Features of ARIA at Maximal Severity by Number of *APOE* ε4 Alleles

eTable 4. Baseline Characteristics of Patients With No ARIA Compared to Asymptomatic or Symptomatic ARIA

eTable 5. Baseline Characteristics of Patients With No ARIA Compared to Asymptomatic or Symptomatic ARIA (Continued)

eTable 6. Rates of Change in the Clinical Dementia Rating Sum of Boxes (CDR-SB)

eAppendix 1. Memory Diagnostic Center Cognitive Battery

eAppendix 2. SAS Code and Model Output for Linear Mixed Models of Change in CDR-SB Over Time

This supplemental material has been provided by the authors to give readers additional information about their work.

© 2025 Paczynski et al. *JAMA Neurology*.

eFigure 1. Representative brain MRI scans from patients with ARIA. ARIA-E is demonstrated on T2 FLAIR images and SWI is used as the heme-sensitive sequence. a) Mild ARIA-E within the right occipital lobe involving sulcus and cortex. b) Moderate ARIA-E with T2 hyperintensities at multiple locations in both cerebral hemispheres, most pronounced in the right frontal lobe. c) Severe ARIA-H, most pronounced within the left hemisphere. d) Mild ARIA-H (single microhemorrhage) within the left frontal lobe. e) Moderate ARIA-H with two foci of superficial siderosis. f) Severe ARIA-H with multiple microhemorrhages in the left occipital cortex.

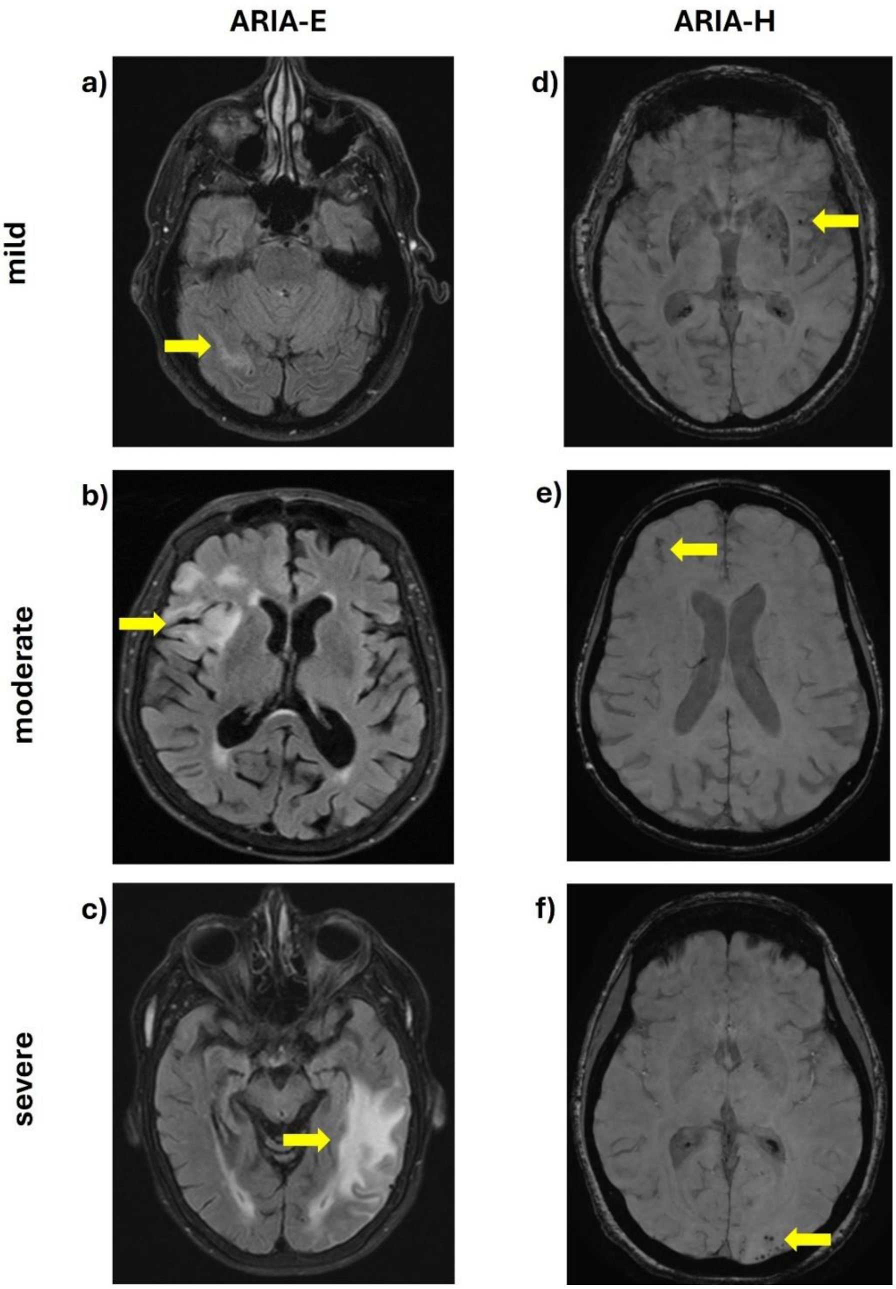

© 2025 Paczynski et al. *JAMA Neurology*.

eFigure 2. Clinical Dementia Rating Sum of Boxes (CDR-SB) over time. The CDR-SB is shown for the baseline visit before lecanemab initiation and the last visit within the study period. Individuals with a baseline CDR of 1 (mild dementia) are shown in purple and those with a baseline CDR of 0.5 (mild cognitive impairment or very mild dementia) are shown in teal. The thick black lines represent the estimated intercept and slope for the CDR 1 (upper line) and CDR 0.5 (lower line) groups. The estimated rate of change was 1.11 CDR-SB/year for the entire group. See **Supplementary Table 6** for slope estimates.

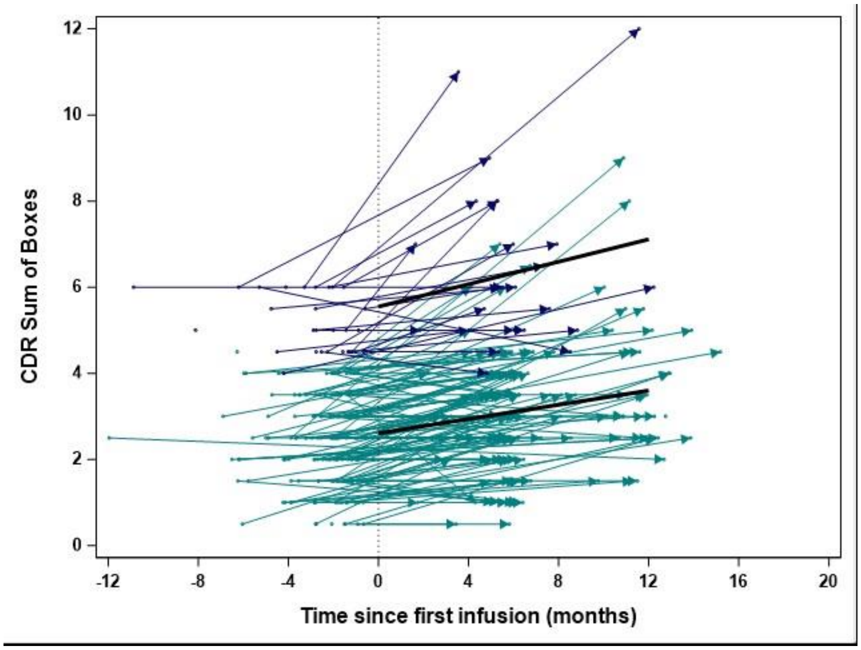

© 2025 Paczynski et al. *JAMA Neurology*.

eTable 1. Characteristics of Memory Diagnostic Center (MDC) patients and CLARITY-AD participants. A total of 3,938 unique patients were seen in the MDC between August 1, 2023 and October 1, 2024: 2,670 had both a CDR and MMSE documented. Characteristics are shown for 1,452 MDC patients who had a documented CDR of 0.5 or 1 and MMSE ≥ 22; 423 MDC patients who had a documented CDR of 0.5 or 1 and MMSE ≥ 22 and biomarker testing for amyloid pathology, and 234 MDC patients treated with lecanemab. For comparison, the characteristics are shown for participants in the CLARITY-AD trial who were treated with lecanemab. The mean value ± standard deviation is shown for continuous variables and the counts with percentages are shown for categorical variables. N.A., not applicable due to missing data. For race and ethnicity, categories may equal >100% due to some participants identifying with multiple groups.

	**MDC patients with CDR 0.5 or 1 and MMSE ≥ 22 n=1,452**	**MDC patients with CDR 0.5 or 1 and MMSE ≥ 22 and biomarker testing n=423**	**MDC patients treated with lecanemab n=234**	**CLARITY-AD participants treated with lecanemab n=859**
**Age (years)**	73.6 ± 8.6	73.2 ± 6.9	74.4 ± 6.7	71.4 ± 7.9
**Sex, n (%) female**	776 (53%)	212 (50%)	117 (50%)	443 (52%)
**MMSE**	26 ± 2	26 ± 2	24 ± 4	26 ± 2
**Clinical Dementia Rating, n (%)**				
0.5	1,217 (84%)	368 (87%)	200 (85%)	694 (81%)
1	235 (16%)	55 (13%)	34 (15%)	165 (19%)
**Race/ethnicity, n (%)**				
White	1,329 (92%)	404 (96%)	229 (98%)	655 (76%)
Black	95 (6.5%)	10 (2.4%)	3 (1.3%)	20 (2.3%)
Asian	9 (0.6%)	4 (0.9%)	1 (0.4%)	147 (17%)
Hispanic	13 (0.9%)	2 (0.5%)	3 (1.3%)	107 (12%)
More than one race	2 (0.1%)	0 (0%)	0 (0%)	0 (0%)
Pacific islander	1 (0.1%)	0 (0%)	0 (0%)	0 (0%)
Declined or missing	16 (1.1%)	5 (1.2%)	1 (0.4%)	37 (4.3%)
***APOE* ε4 genotype, n (%)**				
ε4 homozygote	N.A.	N.A.	20 (8.5%)	136 (16%)
ε4 heterozygote	N.A.	N.A.	125 (53%)	456 (53%)
ε4 non-carrier	N.A.	N.A.	87 (37%)	267 (31%)
ε4 genotype unknown	N.A.	N.A.	2 (0.9%)	0 (0%)

© 2025 Paczynski et al. *JAMA Neurology*.

eTable 2. Length of treatment for patients at risk for ARIA. Only the 194 individuals who received at least four infusions of lecanemab and underwent at least one safety MRI are included. The length of treatment was the interval from the first and last infusion. The average length of treatment during the study period was 6.5 ± 3.2 months (mean ± standard deviation).

**Time (months)**	**Number of patients**
0-2	24
2-4	22
4-6	42
6-8	37
8-10	36
10-12	29
12-14	4

© 2025 Paczynski et al. *JAMA Neurology*.

eTable 3. Features of ARIA at maximal severity by number of *APOE* ε4 alleles. The significance of differences in ARIA frequency by number of *APOE* ε4 alleles was assessed with Mantel-Haenszel tests. Two patients were missing data on *APOE* genotype and were not included in these analyses.

**Cases of ARIA n (%)**	**All n=192**	***APOE* ε4 non-carrier n=74**	***APOE* ε4 heterozygote n=102**	***APOE* ε4 homozygote n=16**	**p=**
**Any ARIA**	**42 (22%)**	**11 (15%)**	**24 (24%)**	**7 (44%)**	**0.014**
**ARIA-E +/- ARIA-H**	**29 (15%)**	**8 (11%)**	**15 (15%)**	**6 (38%)**	**0.027**
Mild ARIA-E	19 (9.9%)	5 (6.8%)	12 (12%)	2 (13%)	
Moderate ARIA-E	9 (4.7%)	3 (4.1%)	3 (2.9%)	3 (19%)	
Severe ARIA-E	1 (0.5%)	0 (0%)	0 (0%)	1 (6.3%)	
**Isolated ARIA-H**	**13 (6.8%)**	**3 (4.1%)**	**9 (8.8%)**	**1 (6.3%)**	0.37
Mild ARIA-H	12 (6.3%)	3 (4.1%)	9 (8.8%)	0 (0%)	
Moderate ARIA-H	1 (0.5%)	0 (0%)	0 (0%)	1 (6.3%)	
Severe ARIA-H	0 (0%)	0 (0%)	0 (0%)	0 (0%)	
**Symptomatic ARIA**	**11 (5.7%)**	**3 (4.1%)**	**5 (4.9%)**	**3 (19%)**	0.094
Clinically mild	7 (3.6%)	1 (1.4%)	4 (3.9%)	2 (13%)	
Clinically moderate	2 (1.0%)	0 (0%)	1 (1.0%)	1 (6.3%)	
Clinically severe	2 (1.0%)	2 (2.7%)	0 (0%)	0 (0%)	

© 2025 Paczynski et al. *JAMA Neurology*.

eTable 4. Baseline characteristics of patients with no ARIA compared to asymptomatic or symptomatic ARIA. The 194 patients who received at least four lecanemab infusions and one monitoring MRI were considered at risk for ARIA. The significance of the difference between the no ARIA and the ARIA groups was assessed with Student's t-tests for age and MMSE; Mantel-Haenszel tests for *APOE* ε4; and Fisher exact tests for binary or non-ordinal variables. Missing data for race/ethnicity and *APOE* genotype were not included in comparisons. For race and ethnicity, categories may equal >100% due to some participants identifying with multiple groups. N.A., not applicable due to missing data.

	**All at risk for ARIA n=194**	**No ARIA n=152**	**Asymptomatic ARIA n=31**	**p=**	**Symptomatic ARIA n=11**	**p=**
**Age (years)**	74.3 ± 6.7	74.5 ± 6.7	74.6 ± 6.7	0.93	71.3 ± 6.7	0.13
**Sex, n (%) female**	101 (52%)	77 (51%)	16 (52%)	0.99	8 (73%)	0.22
**MMSE**	24 ± 4	24 ± 4	24 ± 4	0.68	19 ± 7	**0.0015**
**Clinical Dementia Rating, n (%)**						
0.5	164 (85%)	131 (86%)	30 (97%)	0.13	3 (27%)	**<0.0001**
1	30 (15%)	21 (14%)	1 (3.2%)		8 (73%)	
**Race/ethnicity, n (%)**						
White	190 (98%)	148 (97%)	31 (100%)	0.68	11 (100%)	0.99
Black	2 (1.0%)	2 (1.3%)	0 (0%)		0 (0%)	
Asian	1 (0.5%)	1 (0.7%)	0 (0%)		0 (0%)	
Hispanic	3 (1.5%)	2 (1.3%)	1 (3%)		0 (0%)	
Unknown	1 (0.5%)	1 (0.7%)	0 (0%)	N.A.	0 (0%)	N.A.
***APOE* ε4 genotype, n (%)**						
ε4 homozygote	16 (8.2%)	9 (6.0%)	4 (13%)	0.052	3 (27%)	0.060
ε4 heterozygote	102 (53%)	78 (52%)	19 (61%)		5 (45%)	
ε4 non-carrier	74 (38%)	63 (42%)	8 (26%)		3 (27%)	
Unknown	2 (1.0%)	2 (1.3%)	0 (0%)	N.A.	0 (0%)	N.A.

© 2025 Paczynski et al. *JAMA Neurology*.

eTable 5. Baseline characteristics of patients with no ARIA compared to asymptomatic or symptomatic ARIA (continued). The 194 patients who received at least four lecanemab infusions and one monitoring MRI were considered at risk for ARIA. The significance of the difference between the no ARIA and the ARIA groups was assessed with Mantel-Haenszel tests for mean arterial blood pressure, microhemorrhage, superficial siderosis and Fazekas ordinal categories; and Fisher exact tests for binary or non-ordinal variables. Missing data for mean arterial blood pressure was not included in comparisons. N.A., not applicable due to missing data.

	All at risk for ARIA n=194	No ARIA n=152	Asymptomatic ARIA n=31	p=	Symptomatic ARIA n=11	p=
**Comorbidities, n (%)**						
Hypertension	119 (61%)	94 (62%)	18 (58%)	0.69	7 (64%)	0.99
Dyslipidemia	161 (83%)	127 (84%)	25 (81%)	0.79	9 (82%)	0.99
Diabetes	21 (11%)	15 (9.9%)	6 (19%)	0.13	0 (0%)	0.60
**Medication use, n (%)**						
Antiplatelet	68 (35%)	53 (35%)	11 (35%)	0.99	4 (36%)	0.99
Anticoagulant	9 (4.6%)	8 (5.3%)	1 (3.2%)	0.99	0 (0%)	0.99
Antihypertensive	81 (42%)	63 (41%)	15 (48%)	0.55	3 (27%)	0.53
**Mean arterial blood pressure (mm Hg)**						
<93	63 (32%)	47 (31%)	13 (42%)	0.59	3 (27%)	0.26
93 to <97	27 (14%)	22 (14%)	5 (16%)		0 (0%)	
97 to <107	68 (35%)	59 (39%)	4 (13%)		5 (45%)	
>107	31 (16%)	20 (13%)	8 (26%)		3 (27%)	
Missing	5 (2.6%)	4 (2.6%)	1 (3.2%)	N.A.	0 (0%)	N.A.
**Baseline number of microhemorrhages, n (%)**						
0	152 (78%)	122 (80%)	24 (77%)	0.53	6 (55%)	**0.015**
1 to 4	39 (20%)	29 (19%)	6 (19%)		4 (36%)	
5 to 9	3 (1.5%)	1 (0.7%)	1 (3.2%)		1 (9.1%)	
**Baseline number of superficial siderosis foci, n (%)**						
0	187 (96%)	148 (97%)	29 (94%)	0.46	10 (91%)	0.40
1	6 (3.1%)	3 (2.0%)	2 (6%)		1 (9.1%)	
2	1 (0.5%)	1 (0.7%)	0 (0%)		0 (0%)	
**Baseline Fazekas score, n (%)**						
0	26 (13%)	20 (13%)	4 (13%)	0.86	2 (18%)	0.33
1	126 (65%)	100 (66%)	22 (71%)		4 (36%)	
2	40 (21%)	31 (20%)	4 (13%)		5 (45%)	
3	2 (1.0%)	1 (0.7%)	1 (3.2%)		0 (0%)	
**Baseline infarcts, n (%)**						
One or more small infarcts (<10 mm)	22 (11%)	14 (9.2%)	5 (16%)	0.33	3 (27%)	0.092
One medium-sized infarct (10-20 mm)	2 (1.0%)	0 (0%)	1 (3.2%)	0.17	1 (9.1%)	0.068

© 2025 Paczynski et al. *JAMA Neurology*.

eTable 6. Rates of change in the Clinical Dementia Rating Sum of Boxes (CDR-SB). The annual rates of change were estimated using the CDR-SB at the baseline visit before lecanemab initiation and the last visit within the study period using linear mixed effects models. The difference between slopes for the CDR 1 and CDR 0.5 groups were not different (p=0.096). **See Supplementary Figure 2** for a plot of the associated data and **Appendix 2** for the code and model outputs.

**Entire group** (n=194 total, n=155 with longitudinal data)			
	Estimate	Standard Error	p=
Intercept	3.0597	0.1070	<0.0001
Slope	1.1058	0.1250	<0.0001

**CDR 1 at baseline** (n=30 total, n=24 with longitudinal data)			
	Estimate	Standard Error	p=
Intercept	5.5493	0.1918	<0.0001
Slope	1.5633	0.3139	<0.0001

**CDR 0.5 at baseline** (n=164 total, n=131 with longitudinal data)			
	Estimate	Standard Error	p=
Intercept	2.6090	0.08163	<0.0001
Slope	0.9920	0.1336	<0.0001

© 2025 Paczynski et al. *JAMA Neurology*.

eAppendix 1. Memory Diagnostic Center cognitive battery. Patients underwent a battery of cognitive tests at most visits that included the following tests: verbal fluency (animal naming within one minute), Boston naming test, Mini Mental State Examination (MMSE), Word List Memory Task, Word List Recall, Short Blessed examination, paragraph recall, Trailmaking A, Trailmaking B, Digit Symbol, Geriatric Depression Screen, clock drawing, brief calculations, and serial 3 subtractions.

© 2025 Paczynski et al. *JAMA Neurology*.

eAppendix 2. SAS code and model output for linear mixed models of change in CDR-SB over time. Individual identifier is “MRN”, Clinical Dementia Rating Sum of Boxes is “CDR_SB”, time from initial lecanemab infusion in years is “INT_INF1y”, baseline CDR global is “BL_CDR”.

Model A: Estimating the intercept and rate of change across all individuals.

```
proc mixed data=CDR_SB;
class MRN; model CDR_SB=INT_INF1y/SOLUTION CL DDFM=satterthwaite;
RANDOM INT INT_INF1/sub=MRN type=un; RUN;
```

Model Information	
**Data Set**	WORK.CDR_SB
**Dependent Variable**	CDR_SB
**Covariance Structure**	Unstructured
**Subject Effect**	MRN
**Estimation Method**	REML
**Residual Variance Method**	Profile
**Fixed Effects SE Method**	Model-Based
**Degrees of Freedom Method**	Satterthwaite

Solution for Fixed Effects

Effect	Estimate	Standard Error	DF	t Value	Pr > |t|	Alpha	Lower	Upper
**Intercept**	3.0597	0.1070	193	28.58	<.0001	0.05	2.8486	3.2708
**INT_INF1y**	1.1058	0.1250	144	8.85	<.0001	0.05	0.8587	1.3529

Type 3 Tests of Fixed Effects

Effect	Num DF	Den DF	F Value	Pr > F
**INT_INF1y**	1	144	78.27	<.0001

© 2025 Paczynski et al. *JAMA Neurology*.

Model B: Estimating the intercept and rate of change in CDR 1 and CDR 0.5 groups.

```
proc mixed data=CDR_SB;
Title 'All individuals, full model with estimates of slopes and intercepts';
class BL_CDR MRN; model CDR_SB=BL_CDR BL_CDR*INT_INF1y/SOLUTION CL
NOINT DDFM=satterthwaite; RANDOM INT INT_INF1/sub=MRN type=un; RUN;
```

Model Information	
**Data Set**	WORK.CDR_SB
**Dependent Variable**	CDR_SB
**Covariance Structure**	Unstructured
**Subject Effect**	MRN
**Estimation Method**	REML
**Residual Variance Method**	Profile
**Fixed Effects SE Method**	Model-Based
**Degrees of Freedom Method**	Satterthwaite

Solution for Fixed Effects									
**Effect**	**BL_CDR**	**Estimate**	**Standard Error**	**DF**	**t Value**	**Pr > |t|**	**Alpha**	**Lower**	**Upper**
**BL_CDR**	1	5.5493	0.1918	189	28.93	<.0001	0.05	5.1709	5.9277
**BL_CDR**	0.5	2.6090	0.08163	189	31.96	<.0001	0.05	2.4480	2.7700
**INT_INF1y*BL_CDR**	1	1.5633	0.3139	157	4.98	<.0001	0.05	0.9432	2.1833
**INT_INF1y*BL_CDR**	0.5	0.9920	0.1336	144	7.43	<.0001	0.05	0.7280	1.2560

Type 3 Tests of Fixed Effects				
**Effect**	**Num DF**	**Den DF**	**F Value**	**Pr > F**
**BL_CDR**	2	189	929.15	<.0001
**INT_INF1y*BL_CDR**	2	150	39.98	<.0001

© 2025 Paczynski et al. *JAMA Neurology*.

Model C: Evaluating for differences in the rate of change in CDR 1 and CDR 0.5 groups.

```
proc mixed data=CDR_SB;
Title 'All individuals, full model for comparing slopes';
class BL_CDR MRN; model CDR_SB=INT_INF1y BL_CDR
BL_CDR*INT_INF1y/SOLUTION CL DDFM=satterthwaite;
RANDOM INT INT_INF1/sub=MRN type=un; RUN;
```

Model Information	
**Data Set**	WORK.CDR_SB
**Dependent Variable**	CDR_SB
**Covariance Structure**	Unstructured
**Subject Effect**	MRN
**Estimation Method**	REML
**Residual Variance Method**	Profile
**Fixed Effects SE Method**	Model-Based
**Degrees of Freedom Method**	Satterthwaite

Solution for Fixed Effects									
**Effect**	**BL_CDR**	**Estimate**	**Standard Error**	**DF**	**t Value**	**Pr > |t|**	**Alpha**	**Lower**	**Upper**
**Intercept**		2.6090	0.08163	189	31.96	<.0001	0.05	2.4480	2.7700
**INT_INF1y**		0.9920	0.1336	144	7.43	<.0001	0.05	0.7280	1.2560
**BL_CDR**	1	2.9403	0.2085	189	14.10	<.0001	0.05	2.5290	3.3515
**BL_CDR**	0.5	0	.	.	.	.	.	.	.
**INT_INF1y*BL_CDR**	1	0.5713	0.3411	155	1.67	0.0960	0.05	-0.1026	1.2452
**INT_INF1y*BL_CDR**	0.5	0	.	.	.	.	.	.	.

Type 3 Tests of Fixed Effects				
**Effect**	**Num DF**	**Den DF**	**F Value**	**Pr > F**
**INT_INF1y**	1	155	56.10	<.0001
**BL_CDR**	1	189	198.89	<.0001
**INT_INF1y*BL_CDR**	1	155	2.80	0.0960

© 2025 Paczynski et al. *JAMA Neurology*.
