# Supplementary material for: Lecanemab Treatment in a Specialty Memory Clinic
Source: JAMA Neurol. 2025 May 12;82(7):655–65. doi: 10.1001/jamaneurol.2025.1232 (PMC12070285; doi:10.1001/jamaneurol.2025.1232)
Supplement: Supplement 2. — Data Sharing Statement [file jamaneurol-e251232-s002.pdf]

Data Sharing Statement

Paczynski. Lecanemab Treatment in a Specialty Memory Clinic. *JAMA Neurol*. Published May 12, 2025. doi:10.1001/jamaneurol.2025.1232

Data
Data available: No

Additional Information
Explanation for why data not available: Because data in this study were from the health records of clinic patients who have not consented to data sharing, individual level clinical data are not readily sharable. However, anonymized data derived from MDC electronic health records can be requested from https://i2db.wustl.edu/consultation-services/. The MDC plans to deposit data from patients who consent into the ALZ-NET registry.
